# Thermal stability and inactivation of hepatitis C virus grown in cell culture

**DOI:** 10.1186/1743-422X-7-40

**Published:** 2010-02-18

**Authors:** Hongshuo Song, Jin Li, Shuang Shi, Ling Yan, Hui Zhuang, Kui Li

**Affiliations:** 1 Department of Microbiology, Peking University Health Science Center, Beijing 100191, China; 2 Department of Molecular Sciences, University of Tennessee Health Science Center, Memphis, Tennessee 38163, USA

## Abstract

**Background:**

Hepatitis C virus (HCV) is a blood-borne flavivirus that infects many millions of people worldwide. Relatively little is known, however, concerning the stability of HCV and reliable procedures for inactivating this virus.

**Methods:**

In the current study, the thermostability of cell culture-derived HCV (HCVcc, JFH-1 strain) under different environmental temperatures (37°C, room temperature, and 4°C) and the ability of heat, UVC light irradiation, and aldehyde and detergent treatments to inactivate HCVcc were evaluated. The infectious titers of treated viral samples were determined by focus-forming unit (FFU) assay using an indirect immunofluorescence assay for HCV NS3 in hepatoma Huh7-25-CD81 cells highly permissive for HCVcc infection. MTT cytotoxicity assay was performed to determine the concentrations of aldehydes or detergents at which they were no longer cytotoxic.

**Results:**

HCVcc in culture medium was found to survive 37°C and room temperature (RT, 25 ± 2°C) for 2 and 16 days, respectively, while the virus was relatively stable at 4°C without drastic loss of infectivity for at least 6 weeks. HCVcc in culture medium was sensitive to heat and could be inactivated in 8 and 4 min when incubated at 60°C and 65°C, respectively. However, at 56°C, 40 min were required to eliminate HCVcc infectivity. Addition of normal human serum to HCVcc did not significantly alter viral stability at RT or its susceptibility to heat. UVC light irradiation (wavelength = 253.7 nm) with an intensity of 450 μW/cm^2 ^efficiently inactivated HCVcc within 2 min. Exposures to formaldehyde, glutaraldehyde, ionic or nonionic detergents all destroyed HCVcc infectivity effectively, regardless of whether the treatments were conducted in the presence of cell culture medium or human serum.

**Conclusions:**

The results provide quantitative evidence for the potential use of a variety of approaches for inactivating HCV. The ability of HCVcc to survive ambient temperatures warrants precautions in handling and disposing of objects and materials that may have been contaminated with HCV.

## Background

Hepatitis C virus (HCV) is a small enveloped, positive-stranded RNA virus classified within the family *Flaviviridae*, genus *Hepacivirus*. HCV affects an estimated 170 million people worldwide and is a global health problem. Unlike most RNA viruses which usually cause acute diseases, HCV establishes life-long, persistent, intrahepatic infections in a majority of infected individuals, leading frequently to the development of cirrhosis and hepatocellular carcinoma [1,2]. Because the current, interferon-based treatment regimens eradicate HCV in only about 50% of patients, prevention of HCV infection is pivotal for controlling this viral pathogen.

HCV is transmitted primarily via percutaneous exposure to infectious blood. Prior to the introduction of anti-HCV screening tests in the early 1990s, receiving blood and blood products or organ transplants was a major risk factor for acquiring HCV infection. Currently, injection of illicit drugs represents a major risk, while other routes of infection, including occupational exposure (such as needle stick), sex, and mother-to-infant transmission (with the exception of HIV-coinfected mother), seem infrequent [3]. Interestingly, it was shown recently in the chimpanzee model that HCV in infectious plasma could survive drying and environmental exposure to room temperature for at least 16 h. This finding has raised the possibility of person-to-person transmission of HCV via blood-contaminated objects and medical devices [4]. Clearly, it is fundamental to quantitatively determine the stability of HCV under environmental conditions and evaluate reliable procedures for inactivating this virus. However, such efforts have been hampered by the lack of an efficient cell culture system and convenient, small animal models for HCV. Although HCV RNA and antigens have been used as indicators for the presence or absence of virus particles, such detection methods do not distinguish between the infectious and inactivated viruses [4-6]. To circumvent this, several related viruses in the family *Flaviviridae *that can be readily cultured in vitro, e.g., bovine viral diarrhoea virus (BVDV, genus *Pestivirus*), have been used as surrogates for HCV to study the inactivation process [7,8]. Although these model viruses show similarity in virion and genome structure to HCV, more relevant systems are still needed to assess the reliable procedures for inactivating HCV.

The recent establishment of an HCV cell culture system based on a particular molecular clone, JFH-1, offers the opportunity of evaluating the inactivation methods for HCV directly [9-12]. Using the Huh7-25-CD81 cell line that is highly susceptible to HCVcc infection [13], the stability of HCVcc (JFH-1 strain) at different environmental temperatures (37°C, room temperature, and 4°C) was assessed in this study. In addition, the efficacy of several commonly used viral inactivation methods, including heat treatment, UVC light irradiation, aldehyde-mediated fixation, and detergent treatments in eliminating HCVcc infectivity were evaluated. The results revealed that all of these methods were able to inactivate HCVcc, provided proper conditions are met.

## Results

### Stability of HCVcc at 37°C, RT, and 4°C

**Figure 1:**
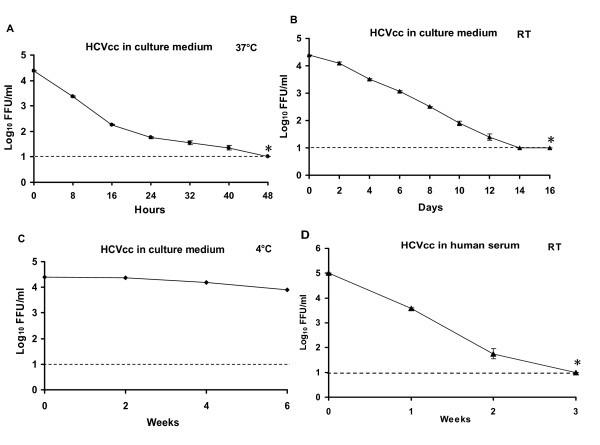
**Stability of HCVcc at 37°C, room temperature (25 ± 2°C), and 4°C**. Aliquots (300 μl) of an HCVcc stock with an initial infectious titer of 2.5 × 10^4 ^FFU/ml were incubated at 37°C (A), room temperature (B), or 4°C (C). In (B) the decay rate of HCVcc infectivity at room temperature was calculated as 0.254-log/day, after the data were fit to a linear regression model (R^2 ^= 0.9963). (D). Aliquots of HCVcc diluted in normal human serum (1.0 × 10^5 ^FFU/ml) were incubated at room temperature. At indicated time points, viral titers were determined by FFU assay on Huh7-25-CD81 cells and shown as mean log_10 _FFU/ml ± SD. The dashed line indicates the detection limit of the FFU assay (10 FFU/ml). The asterisks in (A), (B) and (D) denote time points when infectivity was completely lost (tested negative for HCV NS3 expression to the third cell passage).

To investigate the ability of HCVcc to survive different environmental temperatures, the spontaneous reductions of viral titer at 37°C, RT (25 ± 2°C), and 4°C were determined individually. The HCVcc stock (2.5 × 10^4 ^FFU/ml in culture medium) lost its infectivity after incubation at 37°C for 48 h, when the FFU assay became negative and no residual infectivity was found upon three successive passages of the inoculated Huh7-25-CD81 cultures (Figure 1A). Most of the infectivity loss occurred within the first 24 h [from 2.5 × 10^4 ^FFU/ml to (5.7 ± 0.6) × 10^1 ^FFU/ml, a 2.6-log reduction], while a further 0.4-log reduction in the following 16 h brought the virus titer down to (2.3 ± 0.6) × 10^1 ^FFU/ml, close to the detection limit (10 FFU/ml). In contrast to 37°C, viral titers declined much more slowly and smoothly at RT (Figure 1B). Incubation for every 2 days led to 0.4- to 0.5-log reduction in viral titers until day 14 when infectivity dropped to the level of detection limit. The virus stock became completely devoid of infectivity at day 16 (Figure 1B) and later (data not shown). When incubated at 4°C, no obvious loss of viral infectivity was detected within the first 4 weeks (Figure 1C). However, we noticed a nearly 0.5-log reduction of viral titer after 6 weeks, when the experiments were ended. Lindenbach et al reported that the infectivity of J6/JFH1 HCVcc did not change after three freeze-thaw cycles [10]. Consistent with this, we found no obvious reduction of infectivity even after five cycles of freezing and thawing of the JFH1 HCVcc stock (data not shown). This suggests that HCVcc is relatively insensitive to freeze-thaw manipulation.

To determine whether the presence of human blood affects the stability of HCVcc, a concentrated HCVcc stock was diluted in normal human serum to achieve a titer of 1.0 × 10^5 ^FFU/ml. At RT, this HCVcc-containing serum was found to gradually loose its infectivity in 3 weeks, with 1.4- to 1.7- log reduction in viral titer after every week of incubation (Figure 1D). However, when the HCVcc diluted in human serum (1.0 × 10^5 ^FFU/ml) was dried on the surface of cell culture dishes and incubated at RT for 1 week, no infectivity was detected upon inoculation of the reconstituted serum onto naïve Huh7-25-CD81 cells even after three consecutive cell passages (data not shown). In aggregate, these results suggest that HCVcc is able to survive ambient conditions especially in a liquid environment, and that stability of HCVcc is inversely correlated with temperature.

### Effect of heat treatment on HCVcc infectivity

**Figure 2:**
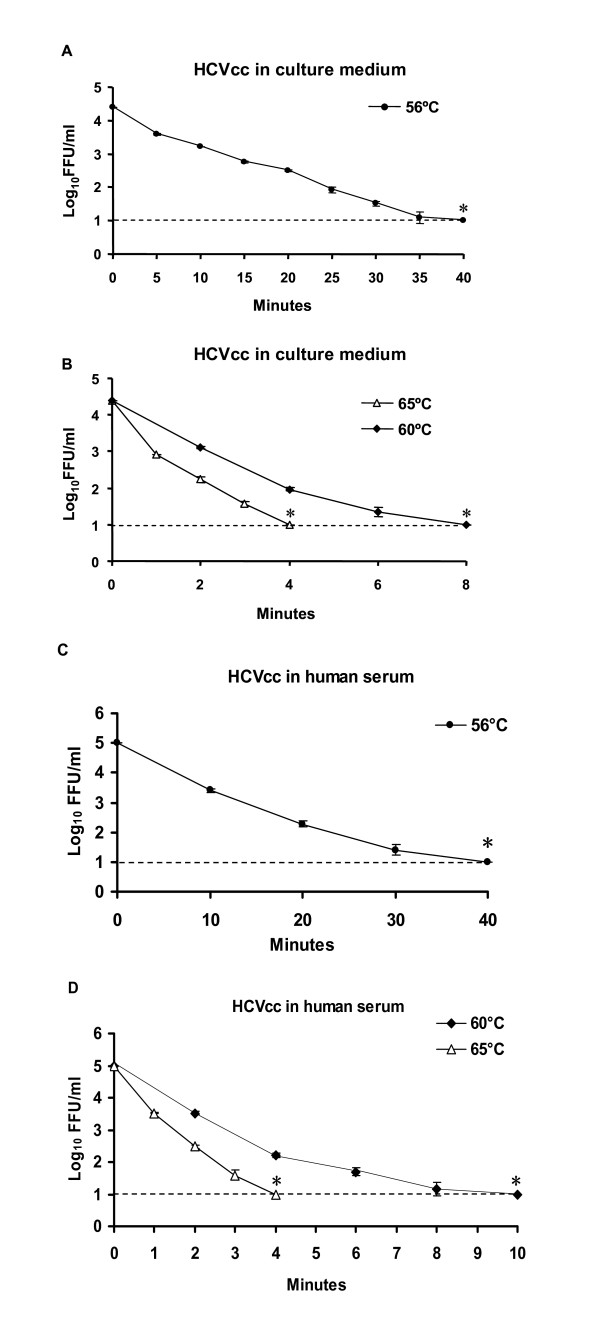
**Inactivation of HCVcc by heat treatment**. Aliquots (100 μl) of an HCVcc virus stock (2.5 × 10^4 ^FFU/ml) or HCVcc diluted in normal human serum (1.0 × 10^5 ^FFU/ml) were incubated in water baths at 56°C (A and C), 60°C and 65°C (B and D). At indicated time points post incubation, samples were removed, cooled on ice-water bath, and their residual infectivity titrated on Huh7-25-CD81 cells. The dashed line indicates the detection limit of the FFU assay (10 FFU/ml). The asterisks denote time points when infectivity was eliminated completely (tested negative in FFU assay and remained negative for HCV NS3 expression to the third cell passage). The decay rate of HCVcc infectivity at 56°C was calculated as 0.089-log/min and 0.119-log/min, for HCVcc in culture medium (A) and human serum (C), respectively, after the data were fit to a linear regression model (R^2 ^= 0.9883 and 0.9813, respectively).

To evaluate the sensitivity of HCVcc to heat treatment, aliquots of HCVcc stock (2.5 × 10^4 ^FFU/ml) were treated with three increasing temperatures (56°C, 60°C, and 65°C, respectively). As shown in Figure 2A, at 56°C HCVcc lost most of its infectivity within 30 min, with a 2.9-log reduction in viral titer [from 2.5 × 10^4 ^FFU/ml to (3.3 ± 0.6) × 10^1 ^FFU/ml). However, after 35 min, a very small amount of infectious virus was still detectable [(1.3 ± 0.6) × 10^1 ^FFU/ml]. Complete viral inactivation took place at 40 min (Figure 2A), and no residual infectivity was detected for samples treated beyond this time point (data not shown). Heat treatments at higher temperatures led to a more rapid decline in viral titer. At 60°C or 65°C (Figure 2B), HCV stocks were inactivated completely by 8 or 4 min, respectively.

Similar kinetics of viral inactivation was observed when heat treatment was performed on HCVcc stocks spiked in human serum. When incubated at 56°C, the viral titer dropped by 3.6-log in the first 30 min [from 1.0 × 10^5 ^FFU/ml to (3.3 ± 0.6) × 10^1 ^FFU/ml]. By 40 min viral infectivity could be no longer detected (Figure 1C). At 60°C and 65°C, the HCVcc-containing serum was completely inactivated by 10 and 4 min, respectively (Figure 2D). Taken together, these results indicate that HCVcc is sensitive to heat treatment and 56°C or higher temperatures could be used for effective HCVcc inactivation. Of note, the presence of human serum does not seem to affect the susceptibility of HCVcc to heat treatment.

### Effect of UVC light irradiation on HCVcc infectivity

**Figure 3:**
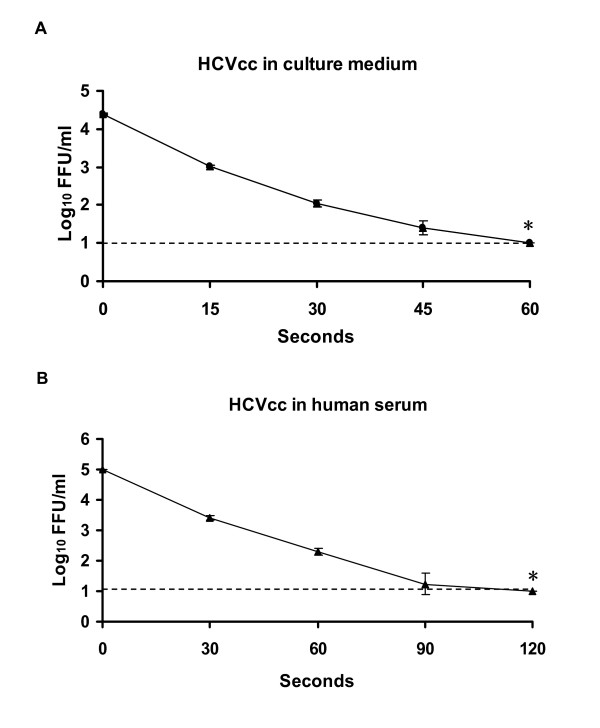
**Inactivation of the HCVcc by UVC light irradiation**. Aliquots (200 μl) of an HCVcc stock (A) or normal human serum containing HCVcc (B) were placed 30 cm beneath the longitudinal midpoint of a UVC lamp. Samples were removed at indicated time points, and viral titers were determined on Huh7-25-CD81 cells immediately. The dashed line indicates the detection limit of the FFU assay (10 FFU/ml). The asterisk denotes the time point when infectivity was completely lost (tested negative in FFU assay and remained negative for HCV NS3 expression to the third cell passage). The decay rate of HCVcc infectivity after UVC light irradiation was calculated as 0.067-log/sec and 0.041-log/sec, for HCVcc in culture medium (A) and human serum (B), respectively, after the data were fit to a linear regression model (R^2 ^= 0.9738 and 0.9891, respectively).

To examine the effect of continuous UVC light on HCVcc infectivity, 200-μl aliquots of HCVcc stock (2.5 × 10^4 ^FFU/ml) were placed in 48-well plates and subjected to UVC light irradiation for different time points, and the residual titers were determined immediately. As shown in Figure 3A, viral titers declined rapidly following UVC irradiation, by 1.4- and 2.4-log, in the first 15 and 30 sec of exposure, respectively. After 45 sec, the viral titer decreased to a level [(2.7 ± 1.2) × 10^1 ^FFU/ml] close to the detection limit of the FFU assay. HCVcc infectivity was eliminated completely after 1 min of irradiation. In contrast, the control, nonirradiated samples incubated at RT for 1 min showed no loss in titer (data not shown).

The effect of UVC light on the infectivity of HCVcc spiked in human serum was also investigated. As shown in Figure 3B, an 1.6-log decrease in viral infectivity was observed after the first 30 sec of irradiation [from 1.0 × 10^5 ^FFU/ml to (2.6 ± 0.5) × 10^3 ^FFU/ml)]. Exposure to UVC light for 90 sec brought the viral titer down to a level that was close to the limit of detection, while an additional 30-sec exposure (a total of120 sec) completely eliminated the residual infectivity (Figure 3B). Given that the radiant intensity at a distance of 30 cm from the UVC lamp was 450 μW/cm^2 ^(where μW = 10^-6 ^J/sec), these data suggest that continuous UVC light at a radiation dose of 5.4 × 10^-2 ^J/cm^2 ^(t = 120 sec) is sufficient to inactivate HCVcc with a titer of 1.0 × 10^5 ^FFU/ml.

### Effects of formaldehyde and glutaraldehyde treatments on HCVcc infectivity

**Figure 4:**
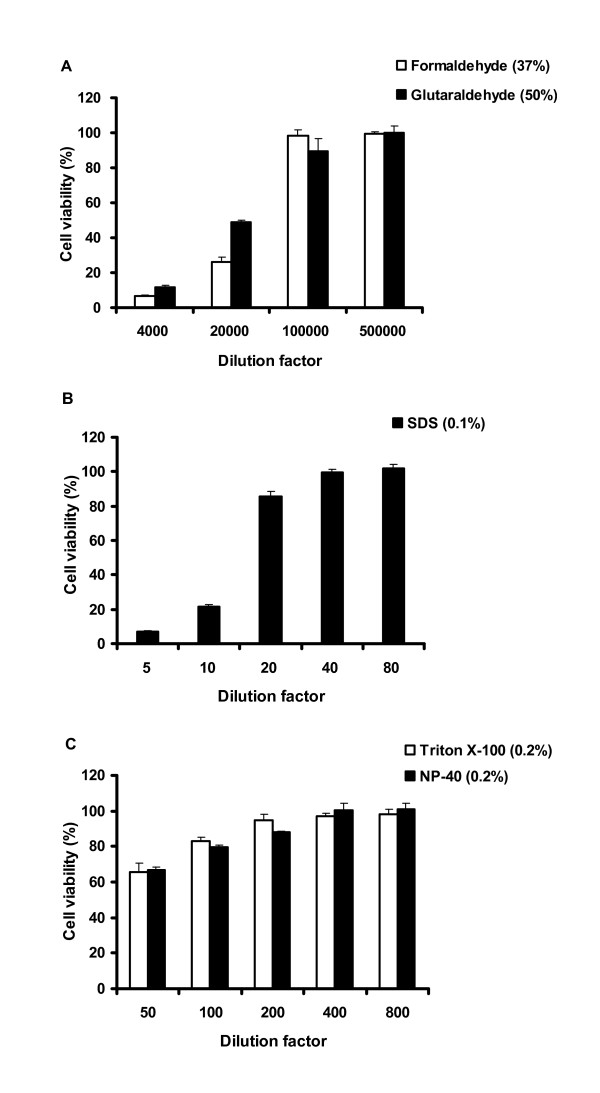
**MTT assay of cytotoxicity associated with various concentrations of aldehydes or detergents**. Solutions of formaldehyde (37% or glutaraldehyde (50%) (A), SDS (0.1%) (B), and Triton X-100 (0.2%) or NP-40 (0.2%) (C) were diluted serially in cell culture medium by dilution factors indicated in the X-axes, then added to Huh7-25-CD81 cells seeded in 96-well plates. The treated cells were refed with 100 μl of fresh medium after 6 h of incubation. MTT assay was carried out after an additional 72 h as described in Methods. Data are presented as the percentage of cell viability relative to the untreated controls (mean ± SD, n = 3).

MTT assay was first carried out to determine the aldehyde concentrations at which they no longer affected cell viability. It was found that 0.00037% formaldehyde and 0.0001% glutaraldehyde no longer had a demonstrable effect on cell growth/viability (Figure 4A). Also, at these aldehyde concentrations, the viral titration results were not perturbed (Table 1 and 2). Therefore, viral samples treated with 0.037% formaldehyde or 0.01% glutaraldehyde were diluted 100-fold for infectivity assay. The reduction in viral titer of an HCVcc stock (4.1 × 10^4 ^FFU/ml) following exposure to each of the aldehydes is summarized in Table 1. At 2 h posttreatment of formaldehyde, a single fluorescent focus was detected in one of the triplicate wells in the FFU assay, while the other two wells showed negative results. Virus samples treated for 2.5 h were negative in the FFU assay; however, a positive IFA result was observed at the second cell passage, indicating the presence of residual infectious virus. After 3 h of treatment, no residual infectivity could be detected up to the third cell passage (Table 1). Compared with 0.037% formaldehyde, 0.01% glutaraldehyde exhibited a higher efficacy in viral inactivation. Ten minutes of glutaraldehyde treatment resulted in more than 1-log reduction in infectivity. After 20 min, the virus stock was inactivated completely, with no residual infectivity being detected up to the third cell passage (Table 1).

**Table 1:** Effects of formaldehyde and glutaraldehyde on infectivity of HCVcc in culture medium

Treatment	Infectious titer (FFU/ml)	IFA result	
		
		2^nd ^cell passage	3^rd ^cell passage
**Formaldehyde (0.037%)**			
0 h	(3.9 ± 0.3) × 10^4a^	NT^c^	NT
0.5 h	(1.1 ± 0.1) × 10^4^	NT	NT
1.0 h	(4.0 ± 1.0) × 10^3^	NT	NT
1.5 h	(1.7 ± 0.6) × 10^3^	NT	NT
2.0 h	≤ 1.0 × 10^3b^	positive	NT
2.5 h	< 1.0 × 10^3^	positive	NT
3.0 h	< 1.0 × 10^3^	negative	negative
**Glutaraldehyde (0.01%)**			
0 min	(4.0 ± 0.3) × 10^4a^	NT	NT
10 min	(3.3 ± 0.6) × 10^3^	NT	NT
20 min	< 1.0 × 10^3^	negative	negative

^a ^Untreated viral samples were retitrated in culture medium containing 0.00037% formaldehyde or 0.0001% glutaraldehyde as controls.

^b^One focus was detected in one of the triplicate wells in FFU assay.

^c ^NT, not tested. Samples with positive results in FFU assay or at the second cell passage were not tested for NS3 staining for the third cell passage.

**Table 2:** Effects of formaldehyde and glutaraldehyde on infectivity of HCVcc in human serum

Treatment	Infectious titer (FFU/ml)	IFA result	
		
		2^nd ^cell passage	3^rd ^cell passage
**Formaldehyde (0.037%)**			
0 h	(8.5 ± 0.7) × 10^4a^	NT^b^	NT
1.0 h	(1.9 ± 0.4) × 10^4^	NT	NT
2.0 h	(3.5 ± 0.7) × 10^3^	NT	NT
3.0 h	(1.0 ± 0.0) × 10^3^	NT	NT
4.0 h	< 1.0 × 10^3^	negative	negative
**Glutaraldehyde (0.01%)**			
0 min	(9.5 ± 0.7) × 10^4a^	NT	NT
10 min	(1.8 ± 0.2) × 10^4^	NT	NT
20 min	(2.5 ± 0.7) × 10^3^	NT	NT
30 min	< 1.0 × 10^3^	Positive	NT
40 min	< 1.0 × 10^3^	negative	negative

^a ^Untreated viral samples were retitrated in culture medium containing 0.00037% formaldehyde or 0.0001% glutaraldehyde as controls.

^b ^NT, not tested. Samples with positive results in FFU assay or at the second cell passage were not tested for NS3 staining for the third cell passage.

The effect of aldehydes on the infectivity of HCVcc in human serum (1.0 × 10^5 ^FFU/ml) was also evaluated by using the same procedure as described above. As shown in Table 2, 3 hours of incubation in 0.037% formaldehyde decreased the viral titer to the limit of detection [(1.0 ± 0.0) × 10^3 ^FFU/ml, a 2-log drop]. After 4 hours, no residual infectivity could be detected (Table 2). In agreement with the results for HCVcc in culture medium, 0.01% glutaraldehyde was also more effective than 0.037% formaldehyde in inactivation of HCVcc in human serum. The results of FFU assay became negative after treatment with 0.01% glutaraldehyde for 30 min, although a small amount of infectious virus was detectable at the second cell passage, By 40 min, no residual infectivity could be detected up to the third cell passage, indicating that effective viral inactivation was achieved (Table 2). In all experiments, the control, PBS-treated samples showed no obvious loss of infectivity when titrated in the presence of 0.00037% formaldehyde or 0.0001% glutaraldehyde (data not shown). These results suggest that both of the aldehydes are able to inactivate HCVcc, regardless of the presence of human serum in HCVcc stocks, and that glutaraldehyde is more effective than is formaldehyde.

**Table 3:** Effects of detergent treatments on HCVcc infectivity

Treatment	Infectious titers before treatment (FFU/ml)^a^	FFU assay after treatment	IFA result at the 3^rd ^cell passage
**0.1% SDS**			
HCVcc in culture medium	(4.1 ± 0.2) × 10^4^	negative	negative
HCVcc in human serum	(9.5 ± 0.7) × 10^4^	negative	negative
cell lysates	(5.5 ± 0.1) × 10^5^	negative	negative
**0.2% Triton X-100**			
HCVcc in culture medium	(2.0 ± 0.2) × 10^4^	negative	negative
HCVcc in human serum	(6.0 ± 0.0) × 10^4^	negative	negative
cell lysate	(3.1 ± 0.2) × 10^5^	negative	negative
**0.2% NP-40**			
HCVcc in culture medium	(1.6 ± 0.2) × 10^4^	negative	negative
HCVcc in human serum	(5.5 ± 0.2) × 10^4^	negative	negative
cell lysate	(2.9 ± 0.2) × 10^5^	negative	negative

^a ^Untreated viral samples or intracellular viral infectivity in untreated, HCVcc-infected cell lysates prepared by freeze-and-thaw were retitrated in the presence of 0.001% SDS, 0.0005% Triton X-100 or 0.0005% NP-40, respectively, to keep in line with the final concentration of the detergents in the treated samples during titration.

### Effects of detergent treatments on HCVcc infectivity

To determine the detergent concentrations at which they were no longer cytotoxic to cells, Huh7-25-CD81 cells were treated with individual, serially diluted detergents and subjected to MTT assay to assess cell viability. Based on the MTT assay results (Figure 4B and 4C), HCVcc stocks (4.1 × 10^4 ^FFU/ml) or those spiked in human serum (1.0 × 10^5 ^FFU/ml) were treated with 0.1% SDS, 0.2% Triton X-100, or 0.2% NP-40, and then tested for infectivity at either 100-fold (SDS-treated samples) or 400-fold (Triton X-100- or NP-40-treated samples) dilution. As summarized in Table 3, all of the detergent-treated samples were negative in the FFU assay and demonstrated no residual infectivity upon three consecutive passages of the inoculated cells (Table 3). In contrast, control samples not treated with detergents showed no obvious loss in virus titers.

The effect of detergents on disrupting intracellular HCVcc virions was also evaluated. JFH-1-infected Huh7-25-CD81 cultures (with 100% cells positive for NS3 as determined by immunofluorescence assay) were lysed in each of the detergent solutions (0.1% SDS, 0.2% Triton X-100, or 0.2% NP-40 in PBS, respectively), and the clarified supernatants were tested for infectivity on naïve Huh7-25-CD81 cells at 100-fold (SDS-lysed samples) or 400-fold (Triton X-100- or NP-40-lysed samples) dilution, respectively. No infectivity was detected from any of these cell lysates in three consecutive passages of inoculated cells (Table 3), while the control cell lysate prepared by freezing and thawing in the absence of any detergents retained high infectivity (Table 3). Taken collectively, these results suggest that each of the detergents, at the tested concentration, is highly effective in eliminating the infectivity of both extracellular and intracellular HCVcc particles.

## Discussion

In this study, a detailed analysis was conducted on the stability of HCVcc at various environmental temperatures. Also evaluated was the efficacy of several conventional viral inactivation procedures in eliminating HCVcc infectivity.

It has been shown previously that genotype 1a HCV in infectious plasma could survive drying and environmental exposure to RT for at least 16 h [4]. The results of the current study have demonstrated that JFH-1 virus (genotype 2a) grown in cell culture can survive 37°C and RT for 2 and 16 days, respectively (Figure 1A and 1B). Of note, the stability of JFH1 HCVcc spiked in human serum did not differ much from those in cell culture medium when incubated at RT (Fig. 1D). When stored at 4°C, JFH-1 virus was found to be relatively stable, without drastic loss of titer during the 6-week observation period (Figure 1C). The latter result is in agreement with a previous report dealing with the J6/JFH1 chimeric virus [10]. The ability of HCVcc to survive various environmental temperatures warrants precautions in handling and disposing objects and materials that may have been contaminated with HCV, to minimize the risk of HCV transmission.

Heat treatment is a widely used viral inactivation method that is effective against both enveloped and nonenveloped viruses [14]. The mechanisms of heat-mediated inactivation include denaturation of viral proteins, as well as disassembly of virus particles into noninfectious viral subunits and single proteins [15]. Viruses other than HCV in the family *Flaviviridae *have been shown to be sensitive to heat treatment. Yellow fever virus is routinely inactivated at 56°C for 30 min. At 60°C, BVDV and yellow fever virus have been reported to be inactivated effectively in 30 and 5 min, respectively [8,16]. In the current study, similar kinetics of viral inactivation following heat treatment was observed for both the HCVcc in culture medium and those in human serum. While 10 min at 60°C or 4 min at 65°C was sufficient to eliminate the infectivity of HCVcc, incubation for 40 min was required to achieve complete viral inactivation at 56°C (Figure 2). Therefore, pretreatment of HCV positive sera for 30 min at 56°C may not be absolutely reliable in eliminating their infectivity. However, because the efficiency of heat treatment could be affected by a variety of factors, such as the initial viral titer, protein concentration in virus suspension, as well as the existence of viral aggregates [8,17] the exact temperature and time required for reliable HCV inactivation should be evaluated under each specific condition.

UV light irradiation is another commonly used physical method for viral inactivation. UVC with a wavelength range of 200-280 nm prevents viral replication by inducing formation of pyrimidine dimers in the viral genome [18]. A recent study reported that BVDV, when suspended in PBS, could be inactivated completely by 1.6 J/cm^2 ^UVC light, while viral suspension containing 5% FBS required a higher radiation dose [7]. The current study demonstrated that HCVcc in culture medium (2.5 × 10^4 ^FFU/ml, volume depth of 0.2 cm) could be inactivated completely by UVC irradiation at a dose of 2.7 × 10^-2 ^J/cm^2 ^within 1 min (Figure 3A), while those spiked in human serum (1.0 × 10^5 ^FFU/ml) required an irradiation dose of 5.4 × 10^-2 ^J/cm^2 ^for full inactivation (Figure 3B). Therefore, UVC light irradiation represents a highly effective means for inactivating HCVcc, the efficiency of which is not affected by human serum components that may interact with HCV virons in vivo. However, the irradiation dose required for each specific occasion may depend on the sample volume and its initial viral titer.

As a chemical cross-linking reagent, formaldehyde inactivates viruses primarily by denaturing viral proteins, as well as the nucleic acids [19,20]. Because the immunogenicity of the viral particles can be retained during inactivation, formalin (37% formaldehyde) treatment is the most used technique for preparing inactivated virus vaccines. For tissue fixation for histology or immunohistochemistry, 10% formalin (or 4% paraformaldehyde) is routinely used. Glutaraldehyde is another effective protein cross-linking reagent, mostly used for fixation of tissues for electron microscopy. Although the detailed mechanisms are not entirely clear yet, successful inactivation of many viruses with glutaraldehyde, including hepatitis B virus, human immunodeficiency virus (HIV), and SARS coronavirus, has been reported [18,21,22]. We demonstrated here that at RT, 3 h of exposure to formaldehyde (0.037%) or 20 min of exposure to glutaraldehyde (0.01%), respectively, could reduce HCVcc infectivity from 4.1 × 10^4 ^FFU/ml to undetectable levels (Table 1). At these concentrations both aldehydes were also effective in inactivating HCVcc in the presence of human serum (Table 2). The slightly longer times required (4 h for formaldehyde treated samples and 40 min for glutaraldehyde treated samples, respectively) were most likely attributed to the 2.5-fold higher initial titer of the HCVcc stock tested (1.0 × 10^5 ^FFU/ml). However, a limitation of the current study is that, because of the cytotoxic effect of the aldehydes, the infectivity of viral samples could be analyzed at only the 100-fold dilution, which may somehow have reduced the sensitivity of the assay. It should be noted, however, the routinely used concentrations of aldehydes for fixation purposes (4% for formaldehyde and 2.5% for glutaraldehyde) are far in excess of the ones examined in the current study and, therefore, should be highly efficient in achieving HCV inactivation.

Detergents are highly efficient at disrupting the lipid-enveloped viruses, and solvent/detergent (S/D) treatment is a standard method for inactivating viruses present in human blood products [22]. The effects of both ionic (SDS) and nonionic (Triton X-100 and NP-40) detergents on HCVcc infectivity have been investigated here. All three detergents at the tested concentrations reduced HCVcc infectivity rapidly to undetectable levels (Table 3). Importantly, both intracellular HCVcc and those released into culture fluid could be inactivated by each of these detergents, regardless of the presence of human serum, indicating that components of culture medium, human serum or intracellular proteins did not interfere with the disruptive processes exerted by these detergents. Under current experimental conditions, effective HCVcc inactivation took place immediately after vortex-mixing, rendering it impossible to delineate the kinetics of viral infectivity reduction during the detergent treatment process. This finding is reminiscent of that reported for HIV in a previous study, which demonstrated that HIV-1 spiked in solution containing 1% Triton X-100 was inactivated completely within 1 min [23]. As in the case of the aldehyde-inactivation experiments, the cytotoxic effect of detergents limited the sensitivity of the current assays. Interestingly, although 0.0005% Triton X-100 and 0.0005% NP-40 had no detectable effect on cell viability (Figure 4C), they still lowered HCVcc infectivity by 1.7- to 2.5-fold when the latter was compared with those determined in the presence of 0.001% SDS or without any detergent (Table 3). Most likely, the residual Triton X-100 or NP-40 still had some disruptive effect on virion integrity, which is important for viral infectivity. Alternatively, these detergents may have caused some cell surface alterations at this extremely low concentration that affect the process of HCVcc entry. However, to maintain the sensitivity of the assay, the detergent-treated samples were not diluted further for the infectivity test. Collectively, the robustness and immediate action of detergents in destroying HCVcc infectivity support the use of S/D treatment procedures in eliminating potential HCV contaminations in blood products.

## Conclusions

In summary, results presented in the current study revealed the stability of HCVcc (genotype 2a, JFH-1 strain) under different temperatures and provided quantitative evidence that heat, UVC light irradiation, aldehyde (formaldehyde and glutaraldehyde), and detergent treatments all can be used as effective means for inactivating HCVcc. However, because the stability and resistance of HCV to different inactivation methods may vary from genotype to genotype, and even strain to strain, the optimal method and procedure used for HCV inactivation should be verified under each particular circumstance. We also note that the results of the current study were developed using an in vitro cell culture system based on hepatoma Huh7 cells, which may differ from normal human hepatocytes in supporting HCV infection. Thus, to what extent the procedures described herein can be applied to an in vivo setting awaits further evaluation.

## Methods

### Cell culture and virus stocks

The Huh7-25-CD81 cell line (a generous gift from Dr. Takaji Wakita), a Huh7 cell clone that stably expresses human CD81 [13], was used throughout the experiments. This cell line was chosen because we found it was approximately 1.5- to 2-fold more sensitive for titration of HCVcc infectivity than was the Huh7.5.1 cell line (data not shown). Cells were maintained in DMEM supplemented with 10% fetal bovine serum (Invitrogen), 10 mM HEPES (Invitrogen), and 400 μg/ml G418 (Merck, Germany) at 37°C in 5% CO_2_. To generate JFH-1 virus stocks, cell culture supernatant collected from full-length JFH-1 RNA-transfected Huh7 cells (kindly provided by Dr. Takaji Wakita) was used to infect Huh7-25-CD81 cells grown in T25 flasks at a multiplicity of infection (MOI) of 0.01. The infected cells were passaged at 3-day intervals with 1:3 to 1:4 split ratios into progressively larger culture vessels. At 12 days postinfection, the culture supernatants were harvested, clarified by centrifugation (5 min at 4000 rpm), and stored in aliquots at -70°C as the HCVcc stock. The infectious titer of the virus stock was determined by focus-forming unit (FFU) assay as described immediately below (the infectious titers of the un-concentrated virus stocks used were either 2.5 × 10^4 ^FFU/ml or 4.1 × 10^4 ^FFU/ml in the current study, as specified in each experiment).

To determine the stability of HCVcc and its susceptibility to individual inactivation methods in the presence of human serum, a condition which better mimics circulating HCV virions in vivo, HCVcc stock was first concentrated using the Amicon Ultra-15 device (100,000 NMWL membrane; Millipore) as described previously [11]. The concentrated virus stock (1.1 × 10^6 ^FFU/ml) was then diluted 11-fold in normal human serum that had been heat-inactivated to achieve an infectious titer of 1.0 × 10^5 ^FFU/ml. This human serum containing HCVcc was stored at -70°C in aliquots until use.

### HCV infectivity assay

The infectious titers of virus stocks and treated viral samples were determined by FFU assay, as described previously [24], using an indirect immunofluorescence assay (IFA) for HCV NS3. In brief, 100 μl of 10-fold serially diluted samples (the dilution factors generally ranged from 1:1 to1:1000) were inoculated onto naïve Huh7-25-CD81 cells seeded in 96-well plates 1 day before infection (7000 cells/well). After 6 h of incubation at 37°C, cells were refed with 100 μl fresh medium. Following an additional 72 h, cells were fixed in 4% paraformaldehyde for 30 min at room temperature (RT), blocked for 60 min in a blocking buffer (3% BSA, 0.3% Triton X-100, 10% FBS in PBS), followed by incubation with a polyclonal antibody against HCV NS3 (kindly provided by Dr. Takaji Wakita) at 1:500 dilution. After 2 h incubation at RT, cells were washed extensively with PBS and then incubated with an FITC-conjugated goat anti-rabbit IgG (Beijing Zhongshanjinqiao, China) at 1:100 dilution for 1 h. Following PBS washes, the numbers of fluorescent foci (a focus is defined as a cluster of infected cells immunostained positive for NS3 antigen) per well at appropriate dilutions (generally containing 5 to 100 foci per well) were counted. The infectious titers, expressed as FFU/ml, were calculated from the average foci number of triplicate or duplicate (for samples derived from human serum spiked with HCVcc) wells. The detection limit of the FFU assay was 10 FFU/ml. For samples with infectious titers below the detection limit of the assay, the potential residual infectivity was examined as follows. Naïve Huh7-25-CD81 cells seeded in 96-well plates were inoculated with samples to be tested (100 μl/well). Inoculated cells were passaged at 3-day intervals from one well into three wells at each passage (with a 1:3 split ratio) to allow growth of residual infectious virus. IFA for NS3 were performed on each cell passage. If the IFA results remained negative for three successive cell passages (up to 9 days postinoculation), the tested sample was considered to be inactivated completely.

### Viral stability assays

An HCVcc stock with a titer of 2.5 × 10^4 ^FFU/ml was dispensed into 300-μl aliquots in tightly capped, 1.5-ml microcentrifuge tubes and then incubated at 37°C, RT (25 ± 2°C), and 4°C, respectively, and protected from light. Aliquots incubated at 37°C were removed every 8 h, while those incubated at RT or 4°C were removed every 2 days or every 2 weeks, respectively, and stored at -70°C until virus titration on Huh7-25-CD81 cells. For viral stability assays for HCVcc spiked in normal human serum (1.0 × 10^5 ^FFU/ml), aliquots were incubated at RT and removed every 7 days for titration. All the time points selected in the experiments were designed based on the results of several pilot experiments.

### Heat treatment

An HCVcc stock in culture medium (2.5 × 10^4 ^FFU/ml) or concentrated HCVcc stock diluted in human serum (1.0 × 10^5 ^FFU/ml) was dispensed into 100-μl aliquots in tightly capped, 1.5-ml microcentrifuge tubes and then incubated in water baths with temperatures of 56°C, 60°C, and 65°C, respectively. At designated time points, aliquots were removed, transferred immediately into ice-water bath to stop the effect of heat, and then subjected to FFU assay for virus titration.

### UVC light irradiation

Two hundred-microliter aliquots of an HCVcc stock (2.5 × 10^4 ^FFU/ml) or HCVcc diluted in normal human serum (1.0 × 10^5 ^FFU/ml) were placed in 48-well plates to give a volume depth of about 0.2 cm and then exposed to continuous UVC light 30 cm beneath the longitudinal midpoint of a UVC lamp (model: ZSZ20D, wavelength = 253.7 nm, Beijing Haidian Konghou High Temperature Composite Material Factory, China). At the distance of 30 cm, the radiant intensity of the UVC lamp was 450 μW/cm^2 ^(where μW = 10^-6 ^J/sec), as specified by the manufacturer. After varying lengths of exposure, samples (200 μl) were removed, and their residual infectivity was titrated on Huh7-25-CD81 cells immediately. Control samples were set up in parallel and incubated for the same time period but protected from UVC light.

### MTT cytotoxicity assay

MTT [3-(4, 5-dimethylthiazol-2-yl)-2, 5-diphenyl tetrazolium bromide] cytotoxicity assay was carried out to determine the concentrations of aldehydes or detergents at which they were no longer cytotoxic to Huh7-25-CD81 cell. Solutions of aldehydes (37% formaldehyde and 50% glutaraldehyde) or detergents [sodium dodecyl sulfate (SDS, 0.1%), Triton X-100 (0.2%) or nonidet P-40 (NP-40, 0.2%)] were diluted serially in cell culture medium, respectively (the range of concentrations for each reagent was designed based on the results of pilot experiments). The diluted reagents were then added to Huh7-25-CD81 cells seeded in 96-well plates (7000 cells/well) 1 day before. After 6 h of incubation at 37°C, the treated cells were refed with 100 μl of fresh culture medium to keep the exposure time to the individual reagents the same as that in HCV infectivity assay. Following an additional 72 h, 20 μl of MTT solution (5 mg/ml, Sigma-Aldrich) was added to each well. After a 4-h incubation at 37°C, the MTT solution was removed and replaced with 200 μl of dimethyl sulfoxide (DMSO, Sigma-Aldrich) per well. After the formazan crystals were dissolved by agitation (10 min at RT), the absorbance of solution in each well was measured at 490 nm using an enzyme-linked immunosorbent assay plate reader (Bio-Rad). The percentage of cell viability was calculated as the ratio of absorbance in treated cells compared with that in untreated controls. All experiments were performed in triplicate and repeated twice.

### Formaldehyde and glutaraldehyde treatments

Formaldehyde (37%) or glutaraldehyde (50%) solutions (Beijing Chemical Reagents Company, China) were diluted in PBS at 1:10 (formaldehyde) or 1:50 (glutaraldehyde), respectively, then added to 500-μl viral samples [HCVcc stock in cell culture medium (4.1 × 10^4 ^FFU/ml) or HCVcc-containing human serum (1.0 × 10^5 ^FFU/ml)] to achieve a final concentration of 0.037% (formaldehyde) or 0.01% (glutaraldehyde), respectively. After different time periods at RT, treated samples were diluted 100-fold in culture medium immediately to stop the inactivation reaction, as well as to eliminate the cytotoxic effect of aldehydes in subsequent FFU assays (according to the results of the MTT assay, the presence of 0.00037% formaldehyde or 0.0001% glutaraldehyde had no appreciable effect on the viability of Huh7-25-CD81 cells). Immediately after the dilution, HCV infectivity in samples was titrated by FFU assay in Huh7-25-CD81 cells. Samples showing negative results in FFU assay were subjected to the residual infectivity test, as described in "HCV infectivity assay" As control, PBS was substituted for the aldehydes to treat the virus stocks, which were then diluted 100-fold to infect cells in the presence of either 0.00037% formaldehyde or 0.0001% glutaraldehyde, respectively.

### Detergent treatments

Solutions of 0.5% SDS (w/v), 1% Triton X-100 (v/v), or 1% NP-40 (v/v) (all prepared in PBS) were added to 500-μl aliquots of viral samples [HCVcc stock in cell culture medium (4.1 × 10^4 ^FFU/ml) or normal human serum containing HCVcc (1.0 × 10^5 ^FFU/ml)] to achieve a final concentration of either 0.1% (SDS) or 0.2% (Triton X-100 and NP-40). After a gentle mixing (within 1 min), treated samples were diluted 100-fold (SDS-treated samples) or 400-fold (Triton X-100- or NP-40-treated samples) immediately in culture medium to negate the cytotoxic effect of detergents (based on the MTT assay results, the presence of 0.001% SDS, 0.0005% Triton X-100, or 0.0005% NP-40 had no demonstrable effect on the viability of Huh7-25-CD81 cells), then subjected to FFU assay. Samples with negative FFU assay results were examined for residual infectivity. As control, PBS was used in place of the detergents to treat the virus stocks, which were subsequently diluted either 100- or 400-fold to infect the Huh7-25-CD81 cells in the presence of 0.001% SDS, 0.0005% Triton X-100, or 0.0005% NP-40, respectively.

To assess the ability of detergents to disrupt intracellular HCV, JFH-1 infected Huh7-25-CD81 cell monolayers grown in 24-well plates (approximately 100% of cells stained positive for NS3 at the time of cell lysis as examined by IFA) were detached by trypsin/EDTA and washed extensively with PBS, and cell pellets were resuspended in 50 μl PBS containing 0.1% SDS, or 0.2% Triton X-100, or 0.2% NP-40, respectively (each detergent was disruptive to cells at these concentrations as visualized by microscopy). After centrifugation, the supernatants of cell lysates were diluted 100-fold (SDS-lysed samples) or 400-fold (Triton X-100- or NP-40-lysed samples) in culture medium for infectivity assays. IFA of HCV NS3 was performed on inoculated cells for three consecutive cell passages. As control, infected cells washed extensively with PBS were pelleted and resuspended in 50 μl PBS and lysed by three cycles of freezing and thawing (-70°C to 37°C), and the infectivity of supernatants was titrated on Huh7-25-CD81 cells.

## Competing interests

The authors declare that they have no competing interests.

## Authors' contributions

HZ and KL conceived the study and designed the experiments. HSS, JL, SS and LY carried out the experimental work. HSS, HZ and KL wrote the paper. All Authors have read and approved the final manuscript.
